# A dataset on social media users’ engagement with religious misinformation

**DOI:** 10.1016/j.dib.2023.109439

**Published:** 2023-07-22

**Authors:** Md. Sayeed Al-Zaman, Mridha Md. Shiblee Noman

**Affiliations:** Department of Journalism and Media Studies, Jahangirnagar University, Savar, Dhaka 1342, Bangladesh

**Keywords:** Misinformation, Religion, Communalism, Social media, Facebook, Bangladesh

## Abstract

This dataset comprises Facebook data on five cases of religious misinformation in Bangladesh. To the best of our knowledge, it is the first publicly accessible dataset on Facebook-based religious misinformation in the country, featuring 7350 comments contributed by unique users. As online religious misinformation has been implicated in causing interreligious violence and tension in Bangladesh, this dataset can offer crucial insights into how social media is being exploited to foment such events. It may also help advance policy reform and activism to protect human rights and prevent the spread of religious misinformation.

**Specification Table**

**Table:** 

Subject	Arts & Humanities; Social Sciences
Specific subject area	Humanities (general); Philosophy & Religion; Library and Information Sciences; Media Technology; Sociology
Type of data	Text; Table; Codebook
How the data were acquired	The data was collected from Facebook's public pages using an automated technique. Afterward, it was properly cleaned and stored in a Microsoft Excel file before being coded by two trained coders.
Data format	Raw Data; Filtered, Coded, and Analyzed Data
Description of data collection	We have chosen to focus on five instances of religious misinformation circulated online and examined 962 publicly available Facebook posts on this topic. To ensure a representative sample, we randomly selected 23 posts (2.39%) for analysis and then collected and cleaned 10,862 comments posted in response to these posts [1]. After cleaning the data, we had a final sample of 7,350 comments. Two experienced coders coded these comments, ensuring high intercoder agreement.
Data source location	Facebook's public pages
Data accessibility	Repository name: Mendeley Data Data identification number: 10.17632/5ykks3psks Direct URL to data: https://data.mendeley.com/datasets/5ykks3psks

## Value of the Data

• Bangladesh, the world's eighth most populous and ninth most Facebook-using country [2,3], has around 43.25 million Facebook users, accounting for 32.8% of its population [4]. To our knowledge, our dataset is the first open-access dataset on Facebook-based religious misinformation in Bangladesh.
• The dataset contains comments from Facebook users on five instances of religious misinformation that resulted in significant social unrest, including interreligious violence and tension. Scholars have observed that such incidents fueled by online misinformation have both immediate and long-term consequences for society, including public lynching and minority deprivation [5].
• Populist trends in digital media have accelerated the distortion of information, creating suspicion and controversy and resulting in successful conspiracies [6]. Therefore, this dataset could be an invaluable resource for researchers studying social media's use in instigating religious misinformation and interreligious violence.
• Analyzing this dataset could offer insights into how religious misinformation and political instruments can form symbiotic relationships. Such insights may lead to policy reforms and encourage activism to protect human rights.

## 1 Objective

This dataset is a component of a larger study on the prevalence of online religious misinformation in Bangladesh [7]. The aim is to investigate how social media users engage with religious misinformation. The primary research methodology employs a two-stage exploratory sequential mixed methods approach, where a qualitative design precedes a quantitative design. In the first stage, a smaller dataset is analyzed qualitatively to develop a codebook. In the second stage, the codebook is applied to a larger sample in a quantitative study to verify and extrapolate the qualitative findings. The present dataset is generated as a part of the quantitative study.

## 2 Data Description

The dataset is composed of two sheets: Raw Data and Coded Data. The Raw Data sheet contains 10,862 unfiltered Facebook comments from five selected misinformation events and their posting date and time (for the cases, see Table 1). These comments were posted in response to Facebook posts that contained misinformation. The Coded Data sheet contains 7350 refined comments from the Raw Data sheet, coded based on a pre-determined codebook (see Table 2).

**Table 1:** Details of the data sources.

No.	Incident	Date	Search keywords	Time range	Total posts	Total interactions
1	Nasirnagar violence	30 Oct 2016	“In Nasirnagar”	29 Oct-3 Nov	116	126,618
2	Rangpur violence	10 Nov 2017	“In Rangpur”	9-12 Nov	222	224,297
3	Narail blasphemy protest	5 May 2019	“In Narail”, “insulting Prophet”	4-9 Apr	137	32,439
4	Comilla violence	30 Oct 2020	“Comilla”, “Muradnagar”, “defame”, “insult”, “Facebook”	30 Oct-2 Nov	290	120,585
5	Sunamganj violence	18 Mar 2021	“In Sunamganj”	18-21 Mar	197	222,612

**Table 2:** Codebook for the quantitative analysis.

Themes & Categories	Defining Factors
*Topics*	
Religious	Mention of religious actions (e.g., namaz, puja); sacred religious texts (e.g., the Quran, hadith); religious monuments, institutions, and objects (e.g., mosque, temple); religious ways of life (e.g., prohibitions); religious entities (e.g., clergies); religious revivalism, religious cohesion.
Political	Mention of regional, national, and local politics (e.g., India-Bangladesh relations); political institutions (e.g., political parties, government, governmental wings, and offices, media); law enforcement (e.g., police); political entities (e.g., political leaders, Islamists, seculars); political processes (e.g., policymaking, political corruption); political ideologies (e.g., Hindutva, anti-Hinduism and anti-Islamism, conspiracies).
Radical	Indications of hatred (e.g., racial, ethnic, and religious slurs); mentions and endorsements of actions (e.g., jihad, revenge, punishment, extremism).
Others	This category includes comments that are different from the three defined topics.
*Reactions*	
Positive	Emotional reactions that are considered positive, such as love, interest, serenity, constructive criticism, and suggestion
Negative	Emotional reactions that are considered negative, such as anger, hatred, despise, frustration, mockery, resentment, and irritation.
Others	This category includes comments whose emotional valence could not be properly identified.
*Appraisal*	
Trust	Trust misinformation with or without proper reasoning.
Deny	Deny misinformation with or without proper reasoning.
Doubt	Neither trust nor deny, perhaps due to insufficient evidence and reasoning skills.
None	No indication of misinformation assessment.

All comments are in Bangla (using both Bangla and English alphabets) and English. For better comprehension, we added simple literal translations of the comments. However, we acknowledge that given the size of the data (i.e., more than 600 A4-size pages), we found it difficult to translate all comments flawlessly.

**Fig. 1:**
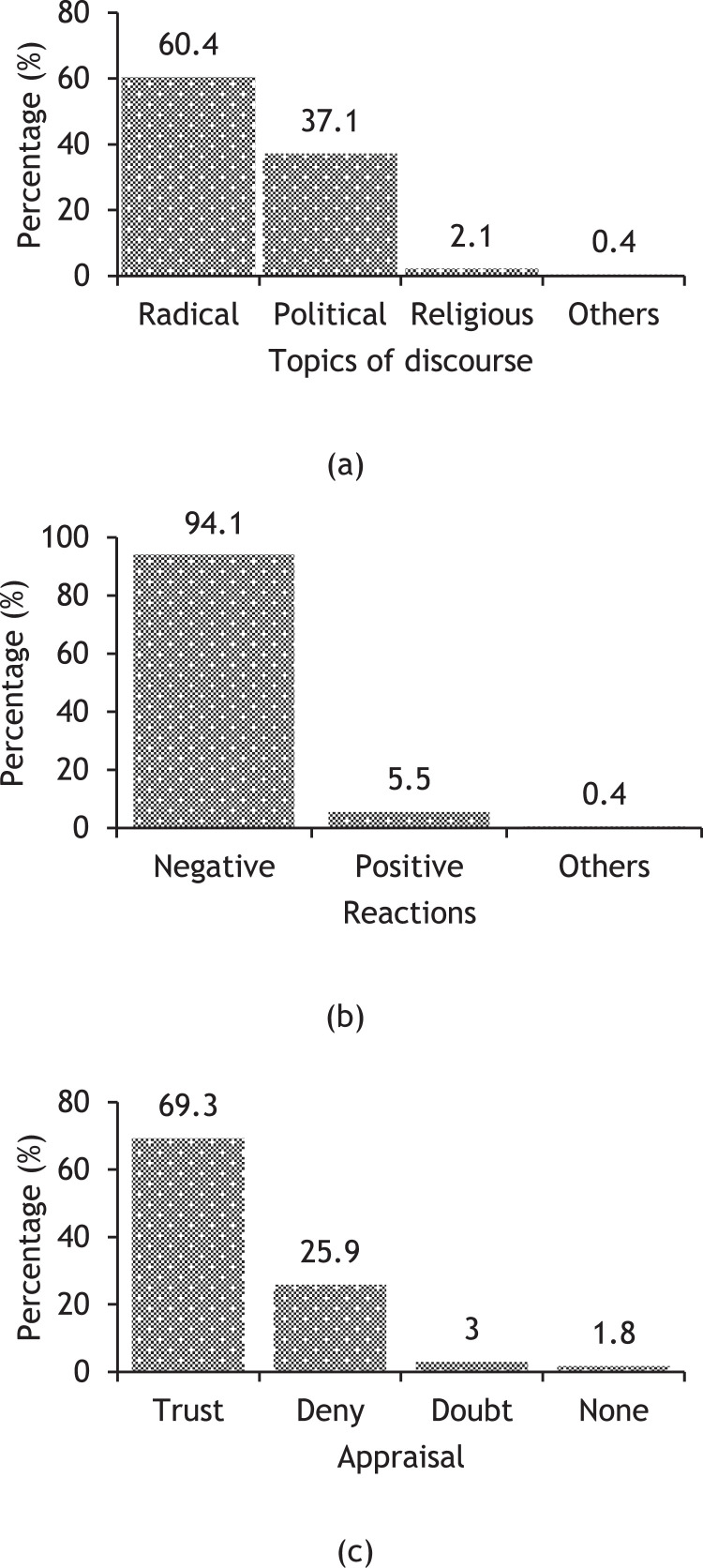
Percentages of users’ various engagement with religious misinformation.

The three columns indicate how social media users engage with religious misinformation: topics, reactions, and appraisals. Each theme contains multiple categories. Fig. 1(a) displays four topic categories. Radical topics (60.4%) are more prevalent in the users’ discussion than political (37.1%) and religious (2.1%). The reactions are coded as negative (94.1%), positive (5.5%), and others (0.4%) (Fig. 1(b)), and the appraisals are coded as trust (69.3%), deny (25.9%), doubt (3%), and none (1.8%) (Fig. 1(c)). Rashid's [8] dataset on Bangla toxic language helped us to a certain extent in determining the valences of reactions.

## 3 Experimental Design, Materials and Methods

We have selected five specific cases to study: the Nasirnagar violence in 2016, the Rangpur violence in 2017, the Narail protest in 2019, the Comilla violence in 2020, and the Sunamganj violence in 2021. While there are other instances of similar misinformation, we have excluded them due to their limited impact and/or online engagement. For instance, the Ramu violence in 2012 was the first interreligious violence driven by misinformation in Bangladesh. However, the incident saw fewer users participating on social media. It could be attributed to two primary factors, among others, such as the lower number of social media users during that period and fewer information sources on Facebook, such as media pages. In all the chosen incidents, the targets were religious minorities, the spread of misinformation was intentional, and the number of casualties was significant.

To search for posts related to each misinformation event, we utilized CrowdTangle [9]. For this purpose, we employed relevant keywords to the events in question (see Table 2). We set the time range to be within 3 to 5 days. Based on our prior experience, we have found that it usually takes less than a week to uncover the truth behind a piece of misinformation. In total, we discovered 962 posts (with a mean (M) of 192.4 and a standard deviation (SD) of 69.52) that either supported or failed to debunk the claims of misinformation. These posts generated 0.73 million interactions (M = 145,310 interactions, SD = 80,490.88).

Out of the 962 Facebook posts, we randomly selected 23 (2.39%) for analysis. These posts generated 10,862 comments, which we collected using Comment Exporter (http://exportcomments.com), a paid data harvesting platform specialized in social media data extraction. In the first step of data cleaning, we eliminated 288 instances of spam, photos, ads, and links. Next, we filtered out 2,015 duplicate comments. Duplicate comments were present in the dataset for two reasons: some users copied and pasted others’ comments instead of creating their own, and the data scraper sometimes used duplicated comments while scraping. Thirdly, we excluded 974 comments made by the same users using their Facebook ID numbers. Finally, after carefully examining the data, we excluded 235 blank, fragmented, or unclear comments. The final corpus contained 7,350 comments from the same number of unique users.

We recruited two trained coders to ensure the accuracy of our data coding process in the quantitative content analysis method. We thoroughly discussed the fundamentals and criteria for coding with them before they began coding the dataset. The unit of analysis for this content analysis was each comment, but we encountered an issue where one comment could contain multiple topics and reactions. To address this problem, we used two approaches. First, we relied on the coders’ subjective interpretation and intuition when coding the comments. Second, we prioritized the dominant meaning conveyed by the comment when assigning codes, which is also subjective. Initially, both coders coded 10% (*n* = 735) of the comments, but we did not achieve an acceptable intercoder agreement.

We held two meetings to discuss the points of disagreement and coding criteria for each variable (i.e., topics, reactions, and appraisal), leading to a third attempt at coding. In this final attempt, we achieved an almost perfect intercoder agreement, as evidenced by Cohen's kappa (κ) values [10]: 0.930 for topics (*p* < 0.05), 0.911 for reactions (*p* < 0.05), and 0.906 for appraisals (*p* < 0.05). These values suggest that our coding is 82-100% reliable [10]. Both coders coded the data independently afterward.

## Ethics Statements

This dataset comprises a large amount of Facebook comments from numerous users, making it unfeasible to obtain individual informed consent. Therefore, following the ethical guidelines of the Association of Internet Researchers [11], we have anonymized all comments in the dataset. Furthermore, we have obtained the misinformation posts using CrowdTangle, a Meta venture that solely collects publicly available data from various social media platforms. Through CrowdTangle, we have legal access to Facebook data. CrowdTangle aims to facilitate researchers’ easy access to public social media content exploration [12]. Apart from that, Facebook's data redistribution policies were also complied with while collecting the data [13].

## CRediT authorship contribution statement

**Md. Sayeed Al-Zaman:** Conceptualization, Methodology, Data curation, Visualization, Formal analysis, Resources, Software, Supervision, Writing – original draft, Writing – review & editing. **Mridha Md. Shiblee Noman:** Writing – original draft, Writing – review & editing.

## Declaration of Competing Interest

The authors declare that they have no known competing financial interests or personal relationships that could have appeared to influence the work reported in this paper.

## Data Availability

Dataset on social media users' engagement with religious misinformation (Original data) (Mendeley Data).
